# A Divergent Selection on Breast Meat Ultimate pH, a Key Factor for Chicken Meat Quality, is Associated With Different Circulating Lipid Profiles

**DOI:** 10.3389/fphys.2022.935868

**Published:** 2022-06-22

**Authors:** Stéphane Beauclercq, Sandrine Mignon-Grasteau, Angélique Petit, Quentin Berger, Antoine Lefèvre, Sonia Métayer-Coustard, Sophie Tesseraud, Patrick Emond, Cécile Berri, Elisabeth Le Bihan-Duval

**Affiliations:** ^1^ INRAE, Université de Tours, BOA, Tours, France; ^2^ Université de Tours, PST Analyse des Systèmes Biologiques, Tours, France; ^3^ UMR 1253, iBrain, Université de Tours, Inserm, Tours, France; ^4^ CHRU de Tours, Service de Médecine Nucléaire In Vitro, Tours, France

**Keywords:** meat quality, ultimate pH, broiler chicken, circulating lipids, targeted lipidomics

## Abstract

**Background:** Chicken meat has become a major source of protein for human consumption. However, the quality of the meat is not yet under control, especially since pH values that are too low or too high are often observed. In an attempt to get a better understanding of the genetic and biochemical determinants of the ultimate pH, two genetic lines of broilers were divergently selected for low (pHu−) or high (pHu+) breast meat pHu. In this study, the serum lipidome of 17-day-old broilers from both lines was screened for pHu markers using liquid-chromatography coupled with mass spectrometry (LC-HRMS).

**Results:** A total of 185 lipids belonging to 4 groups (glycerolipids, glycerophospholipids, sterols, sphingolipids) were identified in the sera of 268 broilers from the pHu lines by targeted lipidomics. The glycerolipids, which are involved in energy storage, were in higher concentration in the blood of pHu− birds. The glycerophospholipids (phosphatidylcholines, phosphatidylethanolamines) with long and polyunsaturated acyl chains were more abundant in pHu+ than in pHu− while the lysophosphatidylcholines and lysophosphatidylethanolamines, known to be associated with starch, were observed in higher quantity in the serum of the pHu− line. Finally, the concentration of the sterols and the ceramides, belonging to the sphingolipids class, were higher in the pHu+ and pHu−, respectively. Furthermore, orthogonal partial least-squares analyses highlighted a set of 68 lipids explaining 77% of the differences between the two broilers lines (R^2^Y = 0.77, Q^2^ = 0.67). Among these lipids, a subset of 40 predictors of the pHu value was identified with a Root Mean Squared Error of Estimation of 0.18 pH unit (R^2^Y = 0.69 and Q^2^ = 0.62). The predictive model of the pHu value was externally validated on 68 birds with a Root Mean Squared Error of Prediction of 0.25 pH unit.

**Conclusion:** The sets of molecules identified will be useful for a better understanding of relationship between serum lipid profile and meat quality, and will contribute to define easily accessible pHu biomarkers on live birds that could be useful in genetic selection.

## Introduction

Poultry meat is mainly consumed as cuts and processed products in most developed countries. For this reason, the technological quality of meat is a major issue for the competitiveness of the poultry meat industry as it affects the storage ability, the processing yields and the organoleptic properties of meat and further processed products (Gigaud et al., 2009; Mir et al., 2017; Baéza et al., 2021). The breast meat ultimate pH (pHu), which is closely linked to muscle glycogen stores (Le Bihan-Duval et al., 2008; Jlali et al., 2012), is one of the most important traits to describe this technological quality (Berri, 2000). The normal pHu range for chicken breast meat is between 5.7 and 6.1. Below 5.7, the meat is referred to as acid, and over 6.1 as DFD (dark, firm, and dry) (Barbut, 1997; Fletcher et al., 2000; Qiao et al., 2001). In rapid-growing chickens, the incidence of these metabolic disorders was estimated about 10 years ago at 18% for acid meat and 5% for DFD meat (Le Bihan-Duval et al., 2020). If the variations in meat pHu result from complex interactions between genetics and rearing factors, a relatively high level of heritability (0.30–0.50) was found for this meat parameter in several genetic lines (Le Bihan-Duval and Berri, 2017). It facilitated the creation of a valuable model of two broiler lines divergently selected on estimated breeding values for breast meat pHu (Alnahhas et al., 2014; Alnahhas et al., 2015). After 14 generations of selection, a deviation of 0.6 pH unit was found between the two lines (Berger et al., 2022). As the level of glycogen stored in breast muscle is also highly heritable (h^2^ = 0.43) and has a −0.97 genetic correlation with pHu (Le Bihan-Duval et al., 2008), this selection strongly modified the glycogen contents of muscles in the divergent lines.

The chickens exhibiting low muscle pHu (pHu− line) were characterized, at 6 weeks of age, by an overexpression of most glycolysis/gluconeogenesis genes and an overabundance of carbohydrates in blood and muscle. The chickens with high meat pHu (pHu+ line) exhibited an overexpression of genes involved in muscle development and of metabolites linked to oxidative stress, muscle proteolysis, and lipid β-oxidation. That was possibly related to an over activation of oxidative energetic metabolism and protein catabolism in response to the glycogen deficiency that characterizes the muscles of the pHu+ line (Beauclercq et al., 2016, 2017). To some extent, the birds of the pHu+ line have metabolic characteristics similar to those described in chickens affected by Wooden Breast myopathy, which also exhibit low muscle glycogen content associated with downregulation of glycolysis and glycogenesis. Alterations in carbohydrate and lipid metabolisms have recently been described as being at the origin of this muscle pathophysiology (Lake and Abasht, 2020). In the case of the pHu+ genetic line, we observed a higher frequency of White Striping, another emerging myopathy, which was associated with an increase in intramuscular fat content at 6 weeks of age (Alnahhas et al., 2016). Compared to the pHu- line, an activation of certain genes involved in lipid metabolism was also reported at this age (Beauclercq et al., 2017). It seems that the divergent selection on the breast meat ultimate pH has also led to variations in lipid metabolism but apart from a lower plasma triglycerides level in the pHu+ chicks at hatch (Métayer-Coustard et al., 2021) or 7 days post-hatching after a challenging start (Bergeot et al., 2022), the circulating lipid profiles of these broilers remain unknown.

High coverage lipidomics has been mostly used in biomedical sciences to develop diagnostic or therapeutic tools based on biomarkers (Hinterwirth et al., 2014; Lu et al., 2019; Wang et al., 2020). However, it has also been used in animal sciences and food sciences to evaluate food quality, to determinate the origin or identify adulteration in fish, milks, oils, and plants (Trivedi et al., 2016; Sun et al., 2020; Kehelpannala et al., 2021). Very recently, muscle lipidome was investigated as well, notably, to differentiate pig breeds (Mi et al., 2019; Zhang et al., 2021) or to study feed efficiency in beef steers (Artegoitia et al., 2019). In poultry, LC-HRMS lipidomics has been used to access the effect of oils in the diet on muscle lipidome (Cui et al., 2020), muscle lipid composition in black-boned silky fowls (Mi et al., 2018; Dirong et al., 2021), or egg yolk composition (Meng et al., 2021; Wood et al., 2021). The blood lipidome has been considered only twice, for one study on heat stress and one on digestive efficiency markers in broilers (Beauclercq et al., 2019; Guo et al., 2021).

This innovative lipidomics study was the first attempt to characterize the serum lipid profiles of broilers selected divergently for high or low breast muscle ultimate pH. The first aim of this study was to know how far this genetic selection for pHu, which impacted the carbohydrate metabolism of birds, also affected lipidic metabolism. The second aim was to identify new potential criteria of selection for meat quality, accessible on live animals. Indeed, meat pH measurement implies sacrificing animals (at 42 days in the conditions of this selection), which means that only a costly sib-selection is feasible. Much gain would be brought by the access to lipidic markers of the pHu available from blood on live animals.

## Materials and Methods

All chemicals were bought from Merck—Sigma Aldrich (Saint-Quentin Fallavier, France) unless otherwise specified.

### Birds and Sample Collection

Chickens originating from the 14th generation of two fast-growing genetic lines divergently selected for high (pHu+) or low (pHu−) ultimate pH of the *Pectoralis major* (*P. major*) muscle were reared at the PEAT experimental unit (https://doi.org/10.15454/1.5572326250887292E12), registered by the French Ministry of Agriculture under license number C-37-175-1 for animal experimentation. The 339 pHu− and 311 pHu+ broilers were fed a 3 phases diet containing a high proportion of sunflower, rapeseed, and faba bean in order to reduce the soybean meal proportion in the diet and were fed and watered *ad libitum* (Berger et al., 2022). Potential interesting feedstuffs, such as Faba bean, sunflower meal and rapeseed were included in the diet based on the results of a former project dedicated to the test of alternative feedstuffs for poultry diets (Pampouille et al., 2021). At 17 days after 6 h of fasting, blood samples for the lipidomics analysis were collected from 134 broilers per line (males and females selected to represent all the diversity of the families within each line), i.e., just before the second diet change and at a time when metabolic differences between the two lines are already established. Indeed, significant differences were seen as early as hatching for muscle glycogen content and 5 days after hatching for ultimate pH (Métayer-Coustard et al., 2021).

After coagulation for 15 min at room temperature and centrifugation (3,000 g for 10 min), sera were aliquoted and stored at −80°C until further analysis. Animals were slaughtered at 42 days and the pHu of the *P. major* muscle was measured the next day after 24 h of chilling, using a portable pH meter (Model 506, Crison Instruments SA, Barcelona, Spain) by direct insertion of the glass electrode into the thickest part of the muscle. Samples were randomly split into a training dataset containing 100 broilers of each line (pHu+: 43 males and 57 females, pHu−: 39 males and 61 females) and an external validation dataset of 34 birds per line (pHu+: 16 males and 18 females, pHu−: 15 males and 19 females). Average pHu values of breast meat were 6.18 ± 0.13 for the pHu+ and 5.57 ± 0.09 for the pHu− in the training data set and 6.20 ± 0.13 for the pHu+ and 5.57 ± 0.08 for the pHu− in the validation data set.

### Lipids Extraction and Mass Spectrometry

LC-HRMS analysis method was adapted from Beauclercq et al*.* (2019). Hundred microliters of serum were combined with 750 µl of a chloroform/methanol mix (1:2), vortexed for 5 s before addition of 250 µl of chloroform and 250 µl of water and vortexed for another 30 s. The resulting mixture was chilled for 20 min at −20°C and centrifuged (15,000 g, 20 min, 4°C). The lower phase was recovered, and put in glass tubes for further solvent evaporation in a SpeedVac at 35°C for around 1 hour. The residues were then reconstituted with 100 µl isopropanol/acetonitrile/water (35:60:5) for Ultra-High-Performance Liquid Chromatography (UHPLC) separation and mass spectrometry analysis. LC-HRMS analysis was performed on a UHPLC Ultimate 3000 system (Dionex, Sunnyvale, CA), coupled to a Q-Exactive mass spectrometer (Thermo Fisher Scientific) and operated in positive (ESI+) and negative (ESI−) ionization modes. Chromatography was carried out with a 1.7 μm XB—C18 (150 mm × 2.10 mm, 100 Å) UHPLC column (Kinetex, Phenomenex, Torrance, CA) heated at 55°C. The solvent system comprised mobile phase A [Isopropanol/Acetonitrile (9:1) + 0.1% (vol/vol) formic acid +10 mM ammonium formate], and mobile phase B [Acetonitrile/Water (6:4) + 0.1% (vol/vol) formic acid +10 mM ammonium formate]; the gradient operated at a flow rate of 0.26 ml/min over a run time of 24 min. The multistep gradient was programmed as follows: 0–1.5 min, 32%–45% A; 1.5–5 min, 45%–52% A; 5–8 min, 52%–58% A; 8–11 min, 58%–66% A; 11–14 min, 66%–70% A; 14–18 min, 70%–75% A; 18–21 min, 75%–97% A; 21–24 min, 97% A. The autosampler (Ultimate WPS-3000 UHPLC system, Dionex, Sunnyvale, CA) temperature was set at 4°C, and the injection volume for each sample was 5 μl. Heated ESI source parameters were a spray voltage of 3.5 kV, capillary temperature of 350°C, heater temperature of 250°C, sheath gas flow of 35 arbitrary units (AU), auxiliary gas flow of 10 AU, spare gas flow of 1 AU, and tube lens voltage of 60 V for C18. During the full-scan acquisition, which ranged from 250 to 1,600 m*/z*, the instrument operated at 35,000 resolution, with an automatic gain control target of 2 × 10^5^ charges and a maximum injection time of 120 ms. The samples were distributed in 2 series. Quality control (QC) samples, a pool of 10 μl of all samples analyzed, were injected at the beginning of both series of analyses, every 10-sample injections, and at the end of both runs.

### Data Processing and Spectral Assignment (Targeted Analysis)

The spectral data acquired in positive and negative ionization modes were processed using XCMS R package implemented in Workflow4Metabolomics tools to extract detected signals and eliminate signal redundancies (Smith et al., 2006; Guitton et al., 2017). The XCMS’ parameters are provided in additional file 1, Supplementary Table S1. The lipids assignment was targeted to an in-house database, with a precision of 5 ppm or 10 ppm for positive or negative ionization modes, respectively. This database was based on the lipidome of several biological matrices from different animals (serum, chicken egg yolk, cells, feces) and contained 542 and 265 lipids in positive and negative ionization mode, respectively. These lipids were annotated with SimLipid software (Premier Biosoft, San Francisco, Ca) according to their retention time, exact mass and MS/MS spectra. The database contains 23 classes of lipids mainly phosphatidylcholine (PC; 279), phosphatidylethanolamine (PE; 91), diacylglycerol (DG; 80), and triglyceride (TG; 61) (additional file 1, Supplementary Figure S1). This annotation reached level 1 on the scale of confidence in lipid identification (Sumner et al., 2007). Lipids were annotated according the LIPID MAPS Lipid Classification System (Fahy et al., 2009). Each peak area was normalized to the total peaks area of each chromatogram. The LC-HRMS analyses were performed in 2 batches. To overcome the signal drift between the 2 batches and be able to compare samples between them, the signal was corrected using the method “LOcally WEighted Scatter-plot Smoother” (LOWESS) available in Workflow4metabolomics tool (Guitton et al., 2017). This method consists in performing a non-linear regression on the QCs, identical in both batches, for each lipid. This regression has then been applied to all samples enabling the comparison of samples from the two batches. Lipids with variability in QC samples greater than 30% were rejected as unsuitable for further investigation. If a lipid was detected both in positive and negative ionization modes, only the data in the positive ionization mode was kept for the subsequent analyses to avoid over-representation of some lipids in the chemometric analysis.

### Chemometric Analysis

#### Orthogonal Projection to Latent Structures Discriminant Analysis and Orthogonal Projection to Latent Structures

An orthogonal projection to latent structures discriminant analysis (OPLS-DA) was performed using the SIMCA 16 Software (version 16.0.2, Umetrics, Umeå, Sweden) on the training dataset (100 pHu+, 100 pHu−). All data were scaled to unit variance. OPLS-DA is a method of supervised classification that predicts the categorical factor Y (pHu+ or pHu− group) by explanatory quantitative variables X (185 lipids). The minimum number of features needed for optimal classification of the OPLS-DA models was determined by iteratively excluding the variables with low regression coefficients and wide confidence intervals derived from jackknifing combined with low variable importance in the projection (VIP) until maximum improvement of the quality of the models. The model quality was evaluated after 7-fold cross validation by cumulative R^2^Y (goodness of fit) and cumulative Q^2^ (goodness of prediction). The contribution of each predictor in the model was evaluated through the variable score contribution (i.e., the differences, in scaled units, for all the terms in the model, between the outlying and the normal observation, multiplied by the absolute value of the normalized weight) and the importance in the model (VIP). Furthermore, the relation between acyl chain properties (i.e., number of carbon atoms, unsaturation) and the pHu lines was studied in each lipid class by plotting the total number of carbons and unsaturation level to the VIP and contribution of each unique lipid as defined by the initial OPLS-DA model. Position jitter was introduced in the lysophosphatidylcholines (LPC), lysophosphatidylethanolamines (LPE), phosphatidylcholines (PC), phosphatidylethanolamines (PE), and sphingomyelin (SM) plots to avoid over plotting. The significance of the differences in number of carbons or unsaturation level between the acyl chains of the lipids contributing to the pHu+ and pHu− lines was tested through Welch’s means equality t-test.

Subsequently, the ability of the lipidome to predict the numerical value of the breast meat pHu on live animals was investigated using OPLS modeling, which differs from OPLS-DA models by the use of a quantitative Y. The pHu values from the chickens included in the training dataset (100 pHu+, 100 pHu−) were fitted to the minimum feature of the lipidome with OPLS. The quality of the model was evaluated by its explicative (R^2^Y) and predictive (Q^2^) abilities, root meaned squared error of estimation (RMSEE), root meaned squared error by a 7-fold cross-validation (RMSEcv), and meaned squared error of prediction (RMSEP) for the external validation.

The OPLS-DA and OPLS models were externally validated on the validation dataset composed of 34 pHu+ and 34 pHu− chickens randomly selected.

The biochemical information about the lipids retained in the OPLS-DA and OPLS models that was used to develop the discussion was partly extracted from the HMDB database (Wishart et al., 2018), Reactome (Jassal et al., 2020), and KEGG (Kanehisa and Goto, 2000).

## Results

### Comparative Lipidic Profiles of the pHu Lines

#### Generation of Lipid Profiles

**FIGURE 1:**
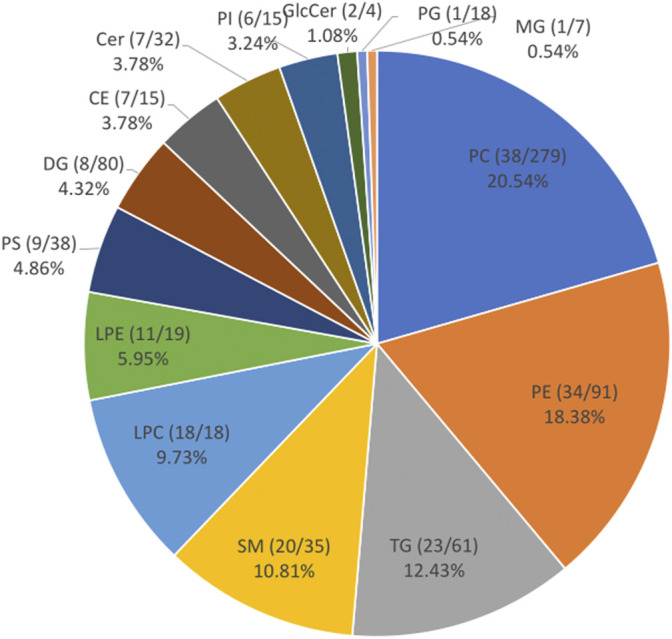
The different classes of lipids identified in the serum of broilers from the pHu lines. The pie chart represents the proportion of each class of lipids in the lipidome of the pHu chickens identified by targeted lipidomics. The names of each lipid class is followed by: the number of lipids in each class/the number of lipids in the class in the database. PC, phosphatidylcholines; PE, phosphatidylethanolamines; PG, phosphatidylglycerols; PI, phosphatidylinositols; PS, phosphatidylserines; SM, Sphingomyelins; TG, triacylglycerols; CE, cholesterol esters; Cer, ceramides; DG, diacylglycerols; GlcCer, glucosylceramides; LPC, lysophosphatidylcholines; LPE, lysophosphatidylethanolamines; MG, monoradylglycerols.

The targeted lipidomics approach chosen for this study permitted the identification of 185 unique lipids (Figure 1) from a database of 731 unique lipids (Supplementary Figure S1). The lipids identified in the sera belong to 14 classes, themselves classified into 4 categories (i.e., glycerolipids, glycerophospholipids, sterols, and sphingolipids). The most represented in our analysis were phosphatidylcholines (PC, 20.54%), phosphatidylethanolamines (PE, 18.38%), triglycerides (TG, 12.43%), sphingomyelins (SM, 10.81%), and lysophosphatidylcholines (LPC, 9.73%).

#### Variation of Lipid Profiles Between the Two pHu Lines

**FIGURE 2:**
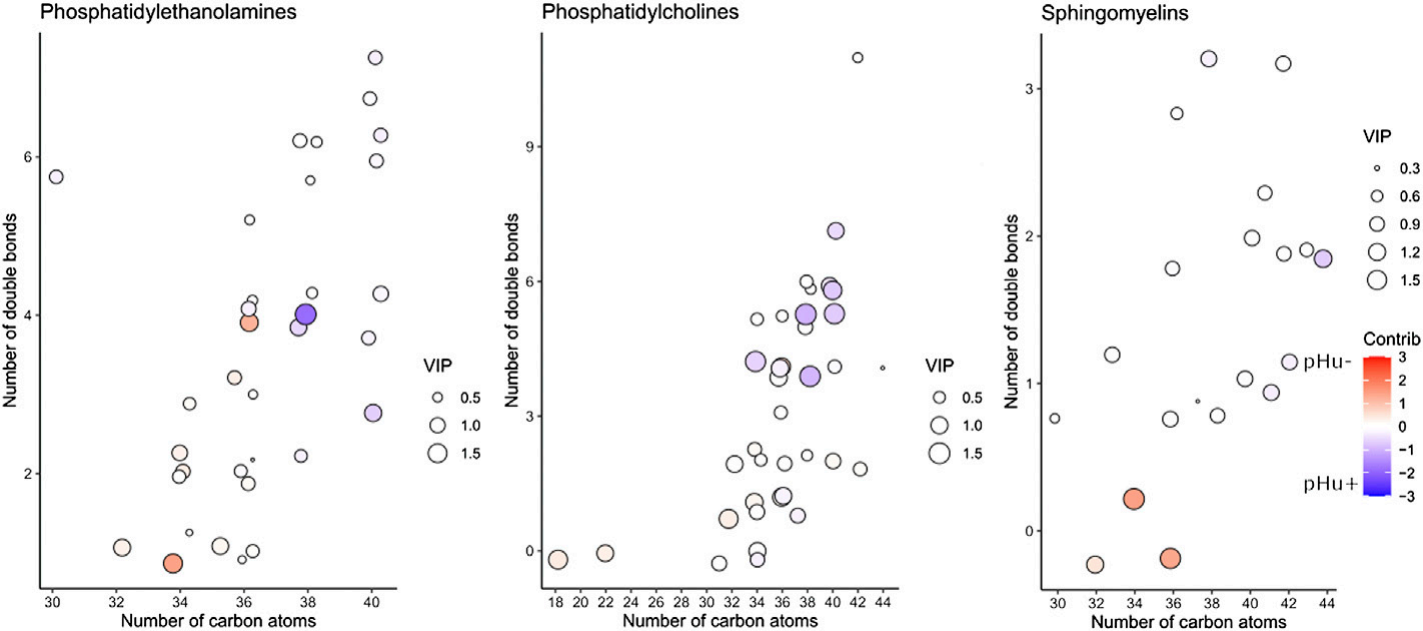
Relation between lipids, acyl chain properties, and the pHu lines. The total number of carbons and unsaturation in the acyl chains of the lipids were plotted with the VIPs and the contributions extracted from the initial OPLS-DA model including the 185 lipids (Supplementary Table S2) identified by targeted lipidomics in the serum of broilers from the pHu lines (training dataset; *n* = 100 for each line). The importance of the lipids (VIP) in the OPLS-DA model (M1a) was represented by the size of the circle and the contribution to the pHu− or pHu+ lines were visualized by a gradient going from red (pHu−) to blue (pHu+). The characteristics of this OPLS-DA model (M1a) are 1 predictive and 3 orthogonal components, R^2^Y_(cum)_ = 0.74 and Q^2^_(cum)_ = 0.56. Position jitter was introduced in the plots to avoid overplotting.

An initial OPLS-DA (M1a) model was fitted to the 185 lipids identified in the sera of the 100 pHu+ and 100 pHu− that compose the training dataset to identify which lipids and acyl chain properties distinguish pHu lines. This model was composed of 1 predictive and 3 orthogonal components and had discriminant [R^2^Y_(cum)_] and predictive [Q^2^_(cum)_] abilities of 0.74 and 0.56, respectively, to separate pHu+ and pHu− lines (Supplementary Table S2). The total number of carbons and unsaturation in the acyl chains of the lipids were plotted and overlaid with the values of the VIPs and the contributions extracted from the OPLS-DA model. Lipids belonging to the LPC, LPE, TG, DG, and Cer classes contributed exclusively or almost exclusively to the pHu-line, Cer containing 36 or 38 carbons in their acyl chain contributing the most (Supplementary Figure S2). The CE and PI were the only two classes of lipids contributing entirely to the pHu+, CE with at least 4 unsaturation contributing the most (Supplementary Figure S2). GlcCer, PG, and Monoacylglycerols (MG) had low contributions. PS, PE, PC, and SM lipid classes differentially contribute to both lines according to their number of carbon atoms and unsaturation in the acyl chains of the lipids (Figure 2). Indeed, the PE with high number of carbons (i.e., ≥ 36; *p*-value = 0.001) and unsaturation (*p*-value = 0.009) in their acyl chains and the PC with high unsaturation number in their acyl chains (*p*-value = 0.005) were more abundant in the serum of the pHu+. Likewise, we observed a tendency (*p*-value = 0.064) for PC with long acyl chains to be also more abundant in the pHu+.

### Fitting Predictive Models of pHu Based on Circulating Lipids

#### Classification Into the Two Genetic Lines

**FIGURE 3:**
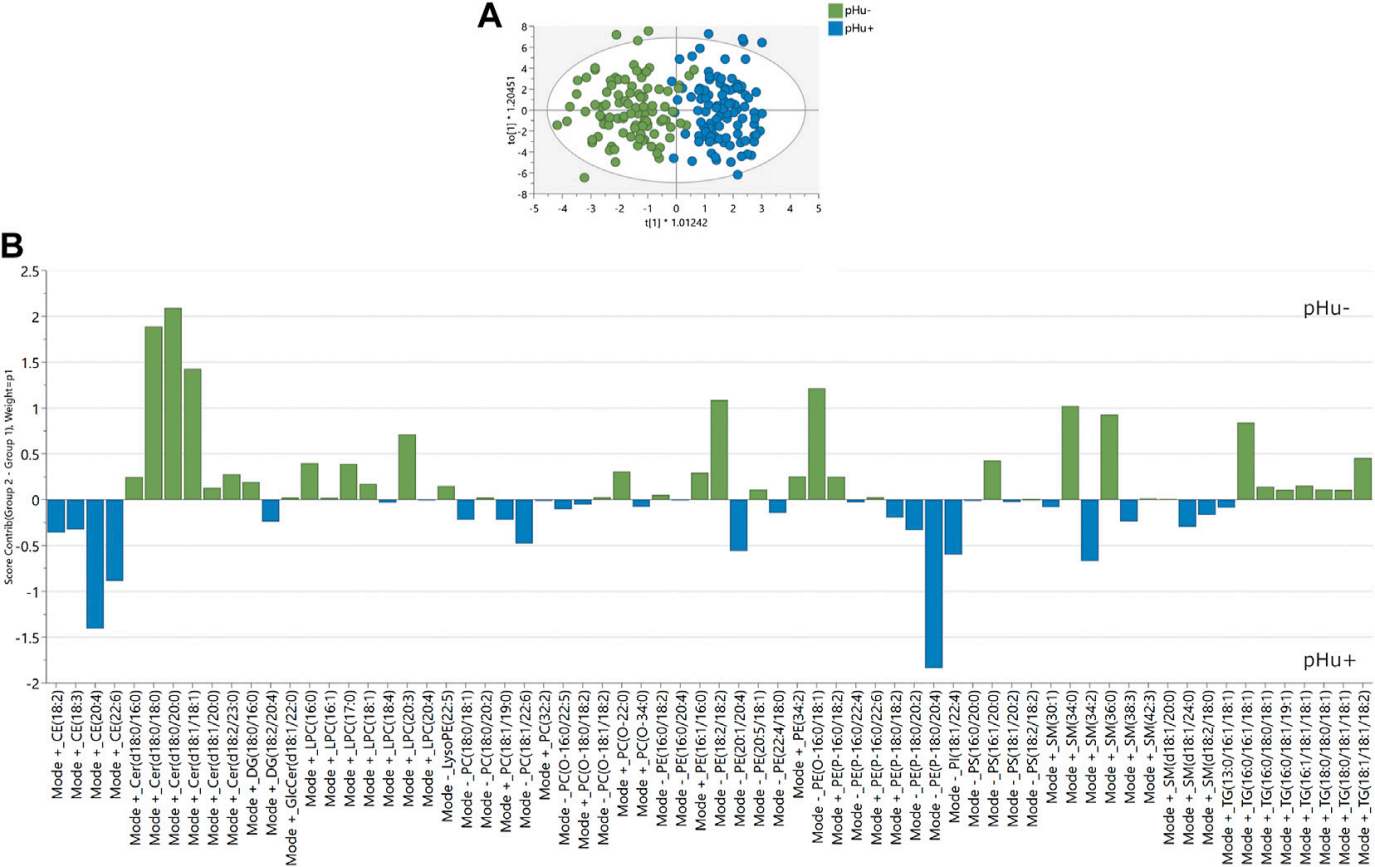
Score and contribution plots following OPLS-DA predictive of the phu line. **(A)** Score plots following OPLS-DA (training dataset) with the chicken lines as categorial factor Y and the lipids as explanatory quantitative variables X. The pHu+ and pHu− individuals were represented by blue and green circles, respectively. The model (M1b) contained 68 lipids, 1 predictive and 3 orthogonal components and its descriptive and predictive performance were R^2^Y_(cum)_ = 0.77 and Q^2^_(cum)_ = 0.67. **(B)** Contribution plot indicating the contribution of the lipids identified in the OPLS-DA predictive of the pHu lines.

Among the 185 lipids identified in the sera of the pHu+ and pHu− lines fitted to the initial OPLS-DA [M1a; R^2^Y_(cum)_ = 0.74, Q^2^_(cum)_ = 0.56], 68 were kept after iteratively excluding the variables with low regression coefficients and wide confidence intervals combined with low VIP until maximum improvement of the quality of the model. Those 68 lipids were included in a new OPLS-DA model (M1b) fitted on 1 predictive and 3 orthogonal components, whose descriptive and predictive performances [R^2^Y_(cum)_ = 0.77 and Q^2^_(cum)_ = 0.67, respectively] were improved in comparison to the model M1a without being over-fitted (Supplementary Figure S3). The score plot (Figure 3A) representing the projection of each chicken tag on the first predictive (x-axis) and first orthogonal (y-axis) components showed that 94% of the pHu− and 97% of the pHu+ were correctly classified by the M1b model. As already observed in the M1a model, the major contributors to the pHu+ belong to the CE and PE classes while the major contributors to the pHu− belong to Cer, LPC, PE, SM, and TG classes (Figure 3B). The performance of the M1b model was tested on the 34 birds from each line, selected randomly to constitute the external validation set. It allows the right classification of 88% of the pHu− and 91% of the pHu+ chickens (Supplementary Figure S4).

#### Prediction of the pHu Phenotype

**FIGURE 4:**
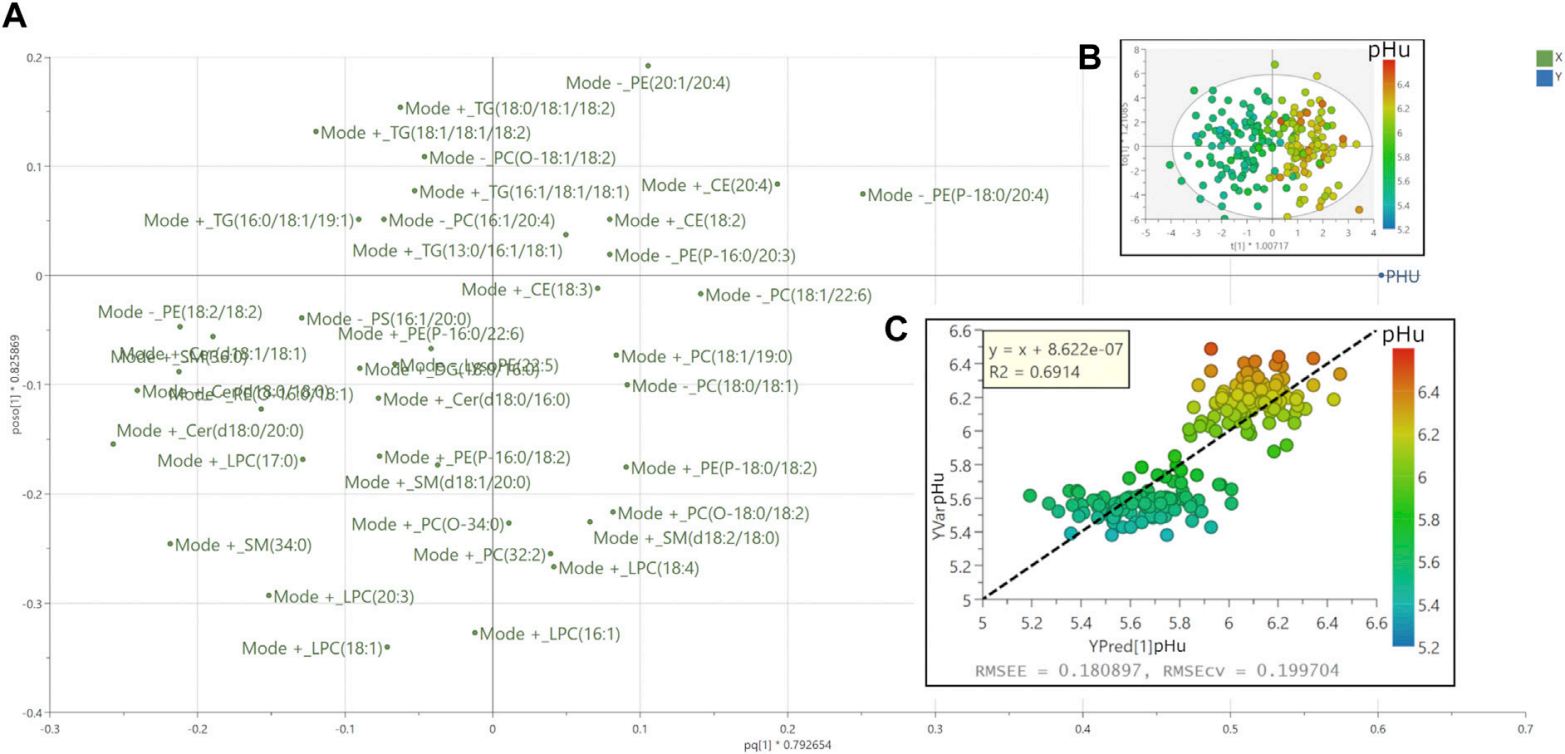
Loadings, scores and observed vs. predicted plots following OPLS model predictive of the pHu value. **(A)** Loadings of the OPLS model (M2; training dataset) with the pHu as quantitative factor Y and the lipids as explanatory quantitative variables X. The model composed of 1 predictive and 2 orthogonal components was fitted to 40 lipids. The cumulative explanatory and predictive performance of this model (M2) were R^2^Y_(cum)_ = 0.69 and Q^2^_(cum)_ = 0.62. **(B)** Scores plots following OPLS. Circles correspond to the chicken tags colored according to their measured pHu value in the *Pectoralis major* muscle. **(C)** Observed vs. predicted after 7-fold cross validation plot. The root mean squared error of the estimation (RMSEE) and the root mean squared error after cross validation (RMSECV) were 0.18 and 0.20 pH unit, respectively.

**FIGURE 5:**
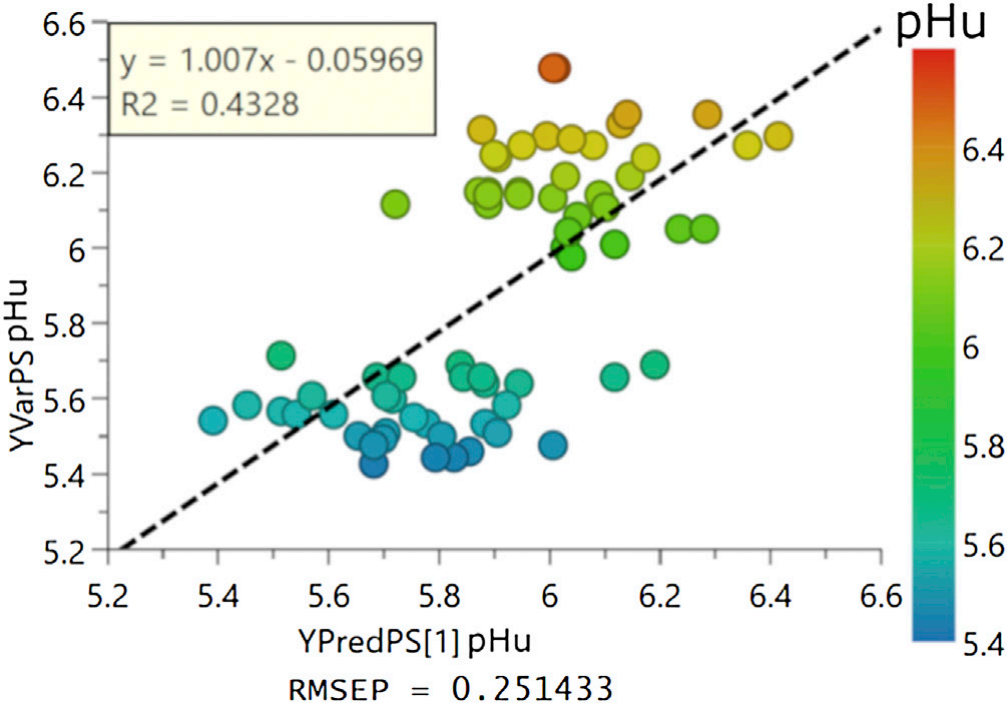
External validation of the OPLS model predictive of the pHu value (M2). Observed vs. predicted plots based on the 34 pHu+ and 34 pHu− broilers selected randomly for external validation. The Root Mean Square Error of Prediction (RMSEP) was 0.25 pH unit.

An OPLS model (M2) was trained to predict breast meat pHu values from a set of serum lipids of the broilers from the training dataset composed of 100 pHu+ and 100 pHu−. This model composed of 1 predictive and 2 orthogonal components was fitted to 40 lipids with a descriptive and predictive abilities of R^2^Y_(cum) _=_ _0.69 and Q^2^_(cum)_ =_ _0.62, respectively. The similarity of the R^2^Y_(cum) _and Q^2^_(cum)_ as well as the permutation plot (Supplementary Figure S5) indicated that the model was not over-fitted. The loadings and scores on the first predictive and orthogonal components were presented in Figures 4A,B. The predictive performance of the model was further assessed by 1) the plotting of the value of the predicted pHu versus the value to the observed pHu and the fitting of a linear regression model to the data and 2) the computing of the RMSEE and RMSEcv. The equation of the regression was Y_obs_ = Y_pred_ + 8.622 × 10^−7^ and its coefficient of determination R^2^ was 0.69, while the RMSEE and RMSEcv were of 0.18 and 0.20 pH unit, respectively (Figure 4C). This model M2 was externally validated on 34 randomly chosen birds from each line. The equation of the linear regression between the pHu observed and predicted in the external validation was Y_obs_ = 1.007 × Ypred - 0.05969 and its R^2^ 0.43 while the RMSEP was 0.25 pH unit (Figure 5).

Thirty-seven of the 40 lipids included in this OPLS model M2 were also included in the OPLS-DA model M1b discriminating the two pHu lines. They were all considered as important in this OPLS (VIP ≥1). Only PC(16:1/20:4), PE (P-16:0/20:3), and TG (18:0/18:1/18:2) were specific to the OPLS model M2 predictive of the pHu value (Supplementary Table S3).

## Discussion

As extensively illustrated by Brown et al. (2022), metabolomic studies are extremely valuable in understanding the physiology of traits of interest for the poultry industry as metabolism is tightly linked to growth or feed efficiency. Metabolomic analyses also allow detecting sometimes unexpected metabolic evolutions linked to genetic selection. In addition, having access to the metabolic signature of the birds from low-invasive biological samples, such as serum, is of particular interest for identifying biomarkers of phenotypes of interest for poultry production or selection. In this study, we considered lipidomics, a subclass of metabolomics methods that measure lipid-based metabolites. It was applied with success in a recent analysis of serum lipidome of chickens divergently selected for digestive efficiency, a pattern of 10 lipids and lipophile compounds being able to explain 82% of the differences of efficiency between the two lines (Beauclercq et al., 2019). Lipidomics also appeared as a relevant approach for exploring the metabolism of the pHu genetic lines. It helped finding a proxy of muscle carbohydrate reserves and chicken-meat quality, knowing that the pHu+ line exhibited a higher lipid percentage in the egg yolk (Petit et al., 2022) but a lower level of plasma triglycerides at hatch (Métayer-Coustard et al., 2021) and one-week of age after a challenging start (Bergeot et al., 2022). Lipidomic profiles were acquired on 17 days aged birds, at a time when daily feed intake is similar between the two lines but feed efficiency is lower in the pHu+ line compared to the pHu− line (Berger et al., 2022).

### Circulating Lipids in Chickens

The circulating lipidome of the broilers analyzed by targeted LC-HRMS lipidomics was composed of 185 lipids belonging to mainly the PC, PE, TG, and SM classes. Those lipid classes were as well the major contributors to the plasma lipidome of mammals, which shows a conserved composition across species (Ishikawa et al., 2015; Kaabia et al., 2018). In birds, most of lipid comparisons were primarily focused on plasma lipoprotein profiles, major lipid components such as total cholesterol, triglycerides and fatty acids, and muscular lipids (Baéza et al., 2015; Mi et al., 2018; Osorio and Flores, 2018). To our knowledge, the only extensive characterization of the circulating lipidome in a bird was done on Quaker parrot (Beaufrère et al., 2020). In this species, the PC and PE were also the major contributors to the serum lipidome with the addition of the fatty acids and conjugates class (FA), PA and PS. By comparison to this untargeted lipidomics study, our lipid database had a similar coverage of most of the lipid classes except the FA, PA, and PI. However, further comparison between the lipidome of those two avian species is limited as their metabolism is most likely very different as it is, for example, the case between mice and rats (Ishikawa et al., 2015). In the same way, a comparison with egg yolk lipid composition, which had been characterized extensively (Meng et al., 2021; Wood et al., 2021), was not relevant.

### Impact of the Divergent Selection on Breast Meat pHu on Blood Lipidome

The lipids identified in the blood of the pHu broilers were from 14 classes (PC, PE, TG, SM, LPC, LPE, PS, DG, CE, Cer, PI, GlcCer, PG, MG) belonging to 4 categories: the glycerolipids, the glycerophospholipids, the sterols, and the sphingolipids. Among those 14 classes of lipids only 12 were kept in the OPLS-DA (M1b) model allowing to separate the pHu+ and pHu-lines.

The pHu-line is characterized by higher concentration of glycerolipids (especially TG and DG) that are involved in energy storage. They are provided by diet or are produced from carbohydrates or glycogen during the lipogenesis which involves the synthesis in liver or adipose tissues of FA from acetyl-CoA and an esterification with glycerol 3-phosphate (Everaert et al., 2021). The higher amounts of glycerolipids observed at 4 weeks of age are consistent with the higher plasma triglyceride levels previously observed in the pHu− at hatch and one-week of age (Métayer-Coustard et al., 2021; Bergeot et al., 2022). Metabolomic characterization of the lines early after hatch and at 6 weeks of age had also suggested that TGs and DGs were preferentially used for energy production by β-oxidation in pHu+ animals, whose energy metabolism was found to be more oxidative than that of pHu−, which themselves have higher carbohydrate stores in the muscle. It is also likely that the low availability of carbohydrate in the pHu+ line may limit TG *de novo* synthesis because *de novo* synthesis of FA requires acetyl-CoA and glycerol 3-phosphate themselves produced through glycolysis.

The pHu lines are also discriminated on their content of certain glycerophospholipids (i.e., PC, PE, PS, PI, PG, LPC, LPE). Glycerophospholipids are the major constituents of the membranes and play roles in signaling cascades. Among them, the PC, also known as lecithins, are the most abundant phospholipids found in cell membranes.

Thirty-eight phosphatidylcholines (PC) were identified in the serum of the pHu broilers, which contribute differentially to the two lines. The PC with high number of unsaturations and long acyl chains were more abundant in the serum of the pHu+, which may suggest in this line a higher activity of the desaturase and elongase during *de novo* synthesis, a greater ability to digest and absorb long PUFA (polyunsaturated fatty acid) or a lower oxidation rate. Indeed, the oxidation rate of medium-chain FA is known to be faster than that of the long-chain fatty acids. In addition, the degree of unsaturation increases the oxidation of FA (Bach et al., 1996; DeLany et al., 2000). The higher concentration of several molecules with antioxidant properties (i.e., betaine, taurine, 1-methylhistidine, and 3-methylhistidine) identified in the pHu+ serum and *Pectoralis major* muscle (Beauclercq et al., 2016) may also exert a protective effect on long-chain PUFA despite a more oxidative energy metabolism in this line (i.e., energy produced from amino acid catabolism and lipid oxidation).

Thirty-four Phosphatidylethanolamine (PE) were identified in the broiler serum, and as for PC, PE with long and polyunsaturated acyl chains were more abundant in pHu+ than in pHu−. Especially, pHu+ were characterized by higher amounts of most of the PE with an (1Z)-alkenyl ether linkage, also referred as neutral plasmalogens. Plasmalogens play a role of antioxidant in addition to their contribution to the decreases in fluidity of the cell membranes and suggested anti-inflammatory properties (Braverman and Moser, 2012; Bozelli et al., 2021). The hydrogen atoms adjacent to the vinyl ether bond have relatively low disassociation energies and are preferentially oxidized when exposed to free radicals. This was proposed to spare the oxidation of polyunsaturated fatty acids and other vulnerable membrane lipids, suggesting a role for plasmalogens as sacrificial oxidants (Braverman and Moser, 2012). The higher serum concentration of plasmogen lipids observed in the pHu+ can reflect a higher adaptive response to oxidative stress due to their propensity for a higher oxidative energy metabolism leading to higher rates of ROS production (Beauclercq et al., 2016).

Lysophosphatidylcholines (LPC) and Lysophosphatidylethanolamine (LPE) also contribute to the definition of the lines. They are products of the partial hydrolysis (i.e., removes one of the FA chain) of PC and PE in the circulation, respectively. In humans, LPC, *via* G protein-coupled receptor signaling, has harmful effects on various cells that include enhancing inflammatory responses, disrupting mitochondrial integrity, and inducing apoptosis (Law et al., 2019). LPC, and LPE to a lesser extent, are lipids also known to be associated with starch (Gayral et al., 2019). For example, lysophospholids represent 84%–94% of the lipids in starch granules comprised of 70% LPC and 20% LPE in oat or wheat (Autio and Eliasson, 2009; Maningat et al., 2009). The higher quantity of LPC and LPE observed in the serum of the pHu− line is therefore consistent with their better feed efficiency (Berger et al., 2022) and potentially greater ability to absorb LPC and LPE associated with starch, even though carbohydrate digestibility has not yet been specifically evaluated in the two lines.

The sterols represented by the cholesterol esters (CE) are essential structural components of cell membranes and also serve as a precursor for the biosynthesis of steroid hormones, bile acid and vitamin D. They are all over-represented in the pHu+ line, especially those with polyunsaturated and longer acyl chain (i.e., > 20), which could be related to very recent observations (personal communication) that evidenced much lower steroid content in egg yolk of pHu+ compared to the pHu−.

Finally, the serum of pHu− appeared richer in several sphingolipids, including Ceramides (Cer), glucosylceramide (GlcCer), and sphingomyelins. Sphingolipids play important roles in signal transduction and cell recognition. Plasma concentrations of sphingolipids have been associated with increased risk of heart failure in humans (Lemaitre et al., 2019) and used to reveal dietary exposure of chickens to fumonisins (Tardieu et al., 2021). Ceramides (Cer) and glucosylceramides (GlcCer) are sphingolipids with a R group consisting of only a hydrogen atom or a glucose molecule, respectively. Sphingomyelins (SM), one of the few membrane phospholipids not synthesized from glycerol, are the conjugation of a PC and a Cer. High serum level of ceramides and sphingomyelins had been associated with the development of obesity, insulin resistance, and impaired glucose metabolism in several studies in human and rodents (Hanamatsu et al., 2014; Turpin et al., 2014; Kayser et al., 2019). Ceramides antagonize insulin signaling by inhibiting transmission of signals through phosphatidylinositol-3 kinase (PI3K) and blocking activation of the kinase Akt/PKB, inhibiting insulin-stimulated glucose uptake in L6 myotubes, and reducing Glut4 translocation in mammals (Sokolowska and Blachnio-Zabielska, 2019), which results in higher glycemia (Kayser et al., 2019). Therefore, the higher concentration of Cer and GlcCer in the serum of the pHu− line is consistent with the higher glycemia of this line that was observed early post-hatch (Métayer-Coustard et al., 2021) and later during growth (Beauclercq et al., 2016).

In addition to the significant differences in muscle glycogen caused by selection on the ultimate pH of the meat, it was observed a higher occurrence and severity of white striping muscle defects, indicative of more fat deposition along muscle fibers, in the pHu+ line (Alnahhas et al., 2016). Recently, it has been hypothesized that excessive lipid accumulation in muscle (as it is observed in case of white striping and wooden breast defects) can cause lipotoxicity and oxidative stress, which may in turn partly lead to the downregulation of glycolysis and glycogenesis and redirection of glucose to alternative utilization pathways (Lake and Abasht, 2020). Indeed, decreased muscle glucose uptake is unlikely to be involved, as some glucose transporters are overexpressed in birds developing wooden breast syndrome. In addition, susceptibility to the wooden breast defect appears to be associated with an overactivation of lipid metabolism evidenced in muscle as early as 2 weeks of age, as shown by the overexpression of genes coding ankyrin repeat domain 1 but also peroxisome proliferator-activated receptors (gamma and alpha) (Lake et al., 2019). Interestingly, we have previously shown that ANKRD1, a gene involved the regulation of lipid metabolism by the peroxisome proliferator-activated receptor alpha, is overexpressed in 6-week-old pHu+ birds muscles (Beauclercq et al., 2017), and has been identified as a biomarker of interest for ultimate meat pH in pigs (Damon et al., 2013). Regarding genes encoding peroxisome proliferator-activated receptors, they were not differentially expressed in the pHu lines (Beauclercq et al., 2017) but PPARG and PPARD are close to SNPs significantly associated with pHu in these lines (Le Bihan-Duval et al., 2018). Taken together, the transcriptomic and metabolomic characterizations of the pHu+ and pHu− lines indicate profound changes in carbohydrate and lipid metabolism related to divergent selection. The present study confirms that these changes are expressed at the muscle and blood levels with the expression of metabolic and lipidomic signatures specific to both lines.

### Development of Predictive Models of the pHu From Blood Lipidome

One of the challenges for the poultry industry, in particular in the field of selection, is to have access to indicators that are available on live animals. The lipidomic characteristics of each line described here thus open interesting perspectives to search for blood indicators of meat pHu, useful to predict this phenotype on reproducers but also to study the impact of nutritional or farming practices on this trait. The lipidomics data acquired on pHu lines served to adjust a model predictive of the pHu value. The predictive ability of the present model was quite similar to a previous one based on muscle transcriptomic data issued of the two lines (Beauclercq et al., 2017). Indeed, the RMSEE, which reports the differences between the values predicted by a model and the observed values, was 0.16 pH units for the model based on the expression in the *P. major* of 20 genes while it was 0.18 pH units in the present model based on 40 circulating lipids. Concretely, the model based on the lipidome allowed us correctly classifying 76% of the muscles exhibiting a normal value of pHu (between 5.7 and 6.1). The lower performance of the external validation (RMSEP = 0.25 pH unit) may result from the lower representation of birds exhibiting a normal value of pHu in the validation dataset. The main interest of the model developed in the present study comes from the possibility of collecting lipidomic data from blood samples taken from live animals, which is especially crucial for selection, and also to obtain a good quality of prediction quite early during the life of the animal (17 days).

## Conclusion

This innovative study is the first attempt at the description of the lipidome of modern broilers and its early variation in relation to the pHu. The focus was set on 4 classes of lipids: the glycerolipids, the glycerophospholipids, the sterols, and the sphingolipids. The glycerolipids, which are involved in energy storage, were in higher concentration in the blood of pHu− birds consistently with their higher glycogen storages. The glycerophospholipids, mainly represented by the phosphatidylcholines and the phosphatidylethanolamines, with long and polyunsaturated acyl chains were more abundant in pHu+ than in pHu− while the lysophosphatidylcholines and lysophosphatidylethanolamines, known to be associated with starch, were observed in higher quantity in the serum of the pHu− line. Finally, the concentration of the sterols and the ceramides, belonging to the sphingolipids class, were higher in the pHu+ and pHu−, respectively. The specific lipidomic blood signatures reported here may help to understand what physiological mechanisms are involved in digestion, transport and metabolic utilization of nutrients, but also, after further validation of the models, to predict the propensity of birds to store more or less glycogen in muscles and the associated pHu.

## Data Availability

The datasets generated and analyzed during the current study are available in the MetaboLights repository hosted by the EMBL-EBI, https://www.ebi.ac.uk/metabolights/MTBLS2970.
