# Supplementary material for: A Divergent Selection on Breast Meat Ultimate pH, a Key Factor for Chicken Meat Quality, is Associated With Different Circulating Lipid Profiles
Source: Front Physiol. 2022 Jun 22;13:935868. doi: 10.3389/fphys.2022.935868 (PMC9257005; doi:10.3389/fphys.2022.935868)
Supplement: Supplementary file 1 [file DataSheet1.PDF]

1	Feature detection (Cent wave)	ppm	10
		snthr	4
		peakwidth	10 50
		mzdiff	0.01
		prefilter peaks	4
		prefilter intensity	30000
		noise	5000
2	Grouping (Density)	bw	5
		mzwid	0.025
		minfrac	0.5
		minsamp	1
3	Retention time correction (obiwarp)	profStep	1
4	Grouping (Density)	bw	5
		mzwid	0.025
		minfrac	0.5
		minsamp	1
5	Filling peaks	/	/
6	CAMERA	annotate	isotopes + adducts
		mzabs	0.015
		ppm	15
		sigma	6
		perfwhm	0.6
		maxcharge	3
		maxiso	5
		intensity	into

Table S1. Parameters used to process the LC-HRMS data (mode + & mode –) with XCMS R package.

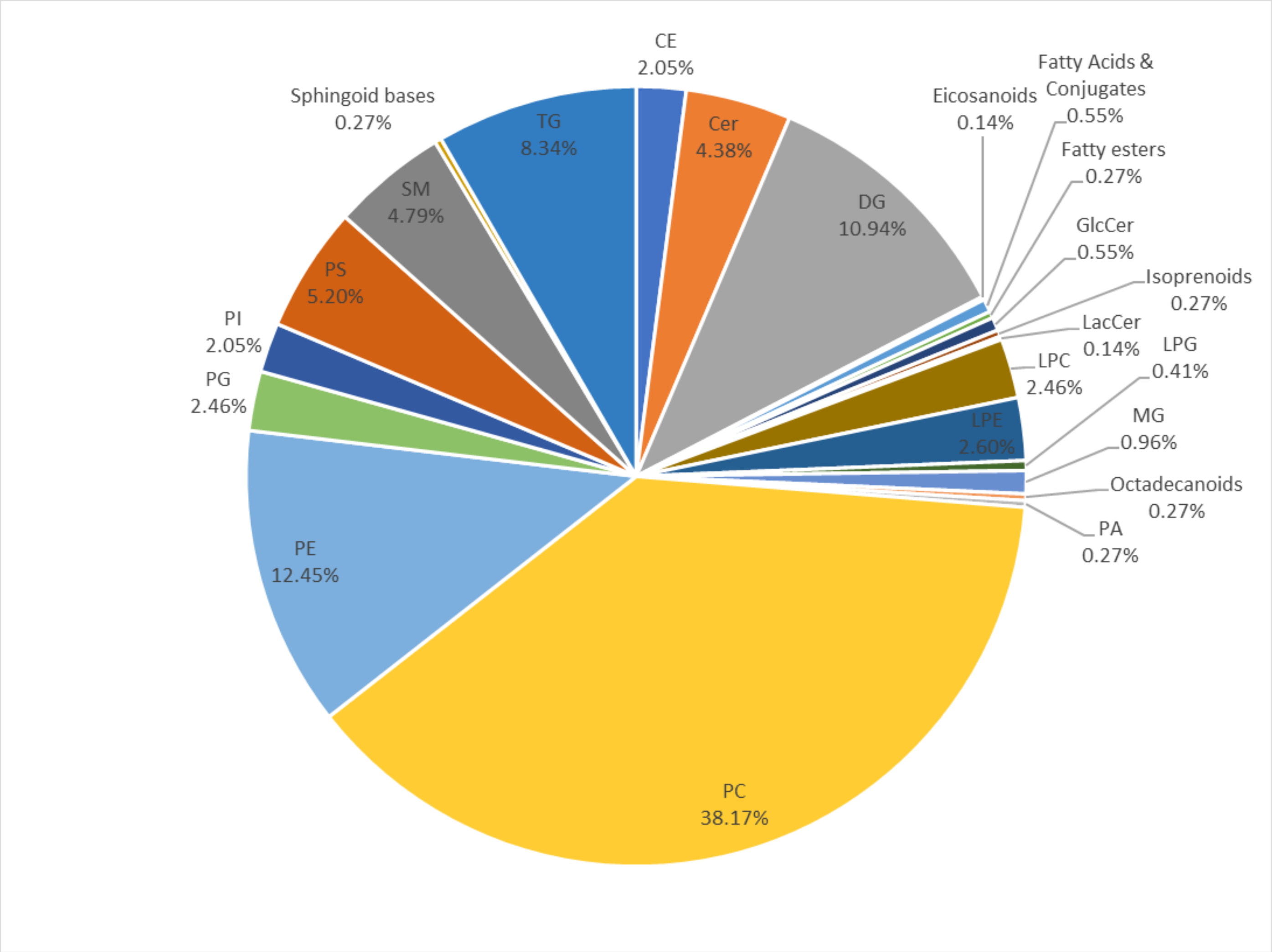

Figure S1. The classes of lipids and the percentage they represent of the background database used for the targeted identification of the lipids in the lipidome of the broilers from the pHu lines. PC = phosphatidylcholines, PE = phosphatidylethanolamines, PG = phosphatidylglycerols, PI = phosphatidylinositols, PS = phosphatidylserines, SM = sphingomyelins, TG = triacylglycerols, CE = cholesterol esters, Cer = ceramides, DG = diacylglycerols, GlcCer = glucosylceramides, LacCer = lactosylceramides, LPC = lysophosphatidylcholines, LPE = lysophosphatidylethanolamine, LPG = lysophosphatidylglycerols, MG = monoradylglycerols, PA = phosphatidic acid.

Lipid ID	Lipid	VIP	Contribution
**Mode +_CE(20:4)**	**CE(20:4)**	1.60485	-1.41302
**Mode +_CE(22:6)**	**CE(22:6)**	1.19543	-0.847395
**Mode +_CE(22:4)**	**CE(22:4)**	1.34957	-0.809949
**Mode +_CE(22:5)**	**CE(22:5)**	1.15542	-0.644901
**Mode +_CE(18:2)**	**CE(18:2)**	0.746702	-0.31989
**Mode +_CE(18:3)**	**CE(18:3)**	0.625889	-0.269798
**Mode +_CE(20:3)**	**CE(20:3)**	0.48525	-0.0611237
**Mode +_Cer(d18:1/20:0)**	**Cer(d18:1/20:0)**	0.700776	0.205177
**Mode +_Cer(d18:2/23:0)**	**Cer(d18:2/23:0)**	0.84626	0.345576
**Mode +_Cer(d18:2/22:0)**	**Cer(d18:2/22:0)**	1.13039	0.379647
**Mode +_Cer(d18:0/16:0)**	**Cer(d18:0/16:0)**	1.11697	0.400732
**Mode +_Cer(d18:1/18:1)**	**Cer(d18:1/18:1)**	1.55366	1.53696
**Mode +_Cer(d18:0/18:0)**	**Cer(d18:0/18:0)**	2.21729	2.54783
**Mode +_Cer(d18:0/20:0)**	**Cer(d18:0/20:0)**	2.29816	2.78842
**Mode +_DG(18:2/20:4)**	**DG(18:2/20:4)**	1.12413	-0.311501
**Mode +_DG(18:0/16:0)**	**DG(18:0/16:0)**	0.687238	0.21838
**Mode +_DG(18:1/18:1)**	**DG(18:1/18:1)**	0.978817	0.240521
**Mode +_DG(16:0/16:0)**	**DG(16:0/16:0)**	0.876594	0.38362
**Mode +_DG(18:2/18:2)**	**DG(18:2/18:2)**	1.32874	0.644907
**Mode +_DG(18:1/16:0)**	**DG(18:1/16:0)**	1.45766	0.891184
**Mode +_DG(16:0/16:1)**	**DG(16:0/16:1)**	1.48967	0.908759
**Mode +_DG(16:1/18:1)**	**DG(16:1/18:1)**	1.50937	0.977413
**Mode +_GlcCer(d18:1/16:0)**	**GlcCer(d18:1/16:0)**	0.434841	-0.0349001
**Mode +_GlcCer(d18:1/22:0)**	**GlcCer(d18:1/22:0)**	0.464922	0.00146593
**Mode +_LPC(18:4)**	**LPC(18:4)**	0.895911	-0.0208428
**Mode +_LPC(20:4)**	**LPC(20:4)**	0.881166	-0.00629754
**Mode +_LPC(22:4)**	**LPC(22:4)**	0.833625	0.00440012
**Mode +_LPC(22:6)**	**LPC(22:6)**	0.725501	0.0048413
**Mode +_LPC(16:1)**	**LPC(16:1)**	1.05932	0.0233722
**Mode +_LPC(18:0)**	**LPC(18:0)**	1.0736	0.11078
**Mode +_LPC(18:1)**	**LPC(18:1)**	1.27489	0.213744
**Mode +_LPC(O-16:1)**	**LPC(O-16:1)**	0.798479	0.232622
**Mode +_LPC(20:5)**	**LPC(20:5)**	1.07783	0.281654
**Mode +_LPC(20:0)**	**LPC(20:0)**	1.17612	0.321337
**Mode +_LPC(O-18:1)**	**LPC(O-18:1)**	1.15028	0.367344
**Mode +_LPC(14:0)**	**LPC(14:0)**	1.27861	0.384708
**Mode +_LPC(18:2)**	**LPC(18:2)**	1.25207	0.394866
**Mode +_LPC(17:0)**	**LPC(17:0)**	1.13631	0.422523
**Mode +_LPC(20:1)**	**LPC(20:1)**	1.36506	0.464815
**Mode +_LPC(16:0)**	**LPC(16:0)**	1.34482	0.496231
**Mode +_LPC(20:2)**	**LPC(20:2)**	1.42774	0.633267
**Mode +_LPC(20:3)**	**LPC(20:3)**	1.58845	0.860857
**Mode +_LPE(22:6)**	**LPE(22:6)**	0.613993	0.0230739
**Mode +_LPE(18:0)**	**LPE(18:0)**	0.903772	0.24393
**Mode +_LPE(18:1)**	**LPE(18:1)**	1.2998	0.403312
**Mode +_LPE(16:1)**	**LPE(16:1)**	1.37544	0.474193
**Mode +_LPE(18:2)**	**LPE(18:2)**	1.25041	0.564654
**Mode +_LPE(16:0)**	**LPE(16:0)**	1.17209	0.568239

**Mode -_LysoPE(20:4)**	**LPE(20:4)**	0.542205	-0.00247706
**Mode -_LysoPE(22:5)**	**LPE(22:5)**	0.951226	0.20762
**Mode -_LysoPE(O-18:1)**	**LPE(O-18:1)**	0.970371	0.294461
**Mode -_LysoPE(P-16:0)**	**LPE(P-16:0)**	0.96976	0.375662
**Mode -_LysoPE(20:3)**	**LPE(20:3)**	1.37483	0.588188
**Mode +_MG(20:4)**	**MG(20:4)**	0.678228	0.0799652
**Mode -_PC(18:1/20:4)**	**PC(18:1/20:4)**	1.51486	-1.00693
**Mode -_PC(18:0/20:4)**	**PC(18:0/20:4)**	1.45861	-0.948961
**Mode -_PC(18:0/22:6)**	**PC(18:0/22:6)**	1.19212	-0.729365
**Mode -_PC(18:0/22:5)**	**PC(18:0/22:5)**	1.41461	-0.700952
**Mode -_PC(14:0/20:4)**	**PC(14:0/20:4)**	1.42198	-0.613477
**Mode -_PC(18:1/22:6)**	**PC(18:1/22:6)**	0.933226	-0.530076
**Mode -_PC(18:1/22:5)**	**PC(18:1/22:5)**	0.960609	-0.408698
**Mode -_PC(16:0/20:4)**	**PC(16:0/20:4)**	1.06056	-0.286002
**Mode -_PC(16:0/18:0)**	**PC(16:0/18:0)**	0.707023	-0.285007
**Mode +_PC(18:1/19:0)**	**PC(18:1/19:0)**	0.774887	-0.249167
**Mode -_PC(18:0/18:1)**	**PC(18:0/18:1)**	0.997108	-0.239979
**Mode +_PC(18:0/22:4)**	**PC(18:0/22:4)**	0.622704	-0.156279
**Mode -_PC(O-16:0/22:5)**	**PC(O-16:0/22:5)**	0.768012	-0.128835
**Mode +_PC(O-36:4)**	**PC(O-36:4)**	1.03707	-0.10062
**Mode -_PC(18:2/20:4)**	**PC(18:2/20:4)**	0.593144	-0.0627586
**Mode +_PC(O-34:0)**	**PC(O-34:0)**	1.00231	-0.0572565
**Mode +_PC(O-18:0/18:2)**	**PC(O-18:0/18:2)**	0.725062	-0.0434571
**Mode -_PC(16:0/22:6)**	**PC(16:0/22:6)**	0.429911	-0.0422702
**Mode +_PC(32:2)**	**PC(32:2)**	0.958231	-0.0175031
**Mode +_PC(42:11)**	**PC(42:11)**	0.373417	-0.00115315
**Mode +_PC(O-38:4)-1**	**PC(O-38:4)-1**	0.663584	-0.00110531
**Mode +_PC(34:5)**	**PC(34:5)**	0.547929	0.00204551
**Mode +_PC(O-44:4)**	**PC(O-44:4)**	0.197956	0.0118019
**Mode -_PC(16:1/18:1)**	**PC(16:1/18:1)**	0.539696	0.0150597
**Mode +_PC(31:0)**	**PC(31:0)**	0.767773	0.0243722
**Mode -_PC(O-18:1/18:2)**	**PC(O-18:1/18:2)**	0.59798	0.0264139
**Mode -_PC(16:0/18:1)**	**PC(16:0/18:1)**	0.792878	0.0268602
**Mode -_PC(18:0/20:2)**	**PC(18:0/20:2)**	0.451444	0.0282559
**Mode -_PC(16:1/20:4)**	**PC(16:1/20:4)**	0.495245	0.115676
**Mode +_PC(42:2)**	**PC(42:2)**	0.650565	0.134323
**Mode +_PC(O-36:1)**	**PC(O-36:1)**	1.12849	0.187446
**Mode +_PC(40:2)**	**PC(40:2)**	0.81709	0.199052
**Mode -_PC(18:0/16:2)**	**PC(18:0/16:2)**	0.671714	0.199297
**Mode +_PC(P-16:0/18:1)**	**PC(P-16:0/18:1)**	1.05675	0.254199
**Mode +_PC(O-22:0)**	**PC(O-22:0)**	0.972317	0.332785
**Mode -_PC(16:0/16:1)**	**PC(16:0/16:1)**	1.23642	0.342025
**Mode +_PC(O-18:0)**	**PC(O-18:0)**	1.25076	0.387825
**Mode -_PC(18:2/18:2)**	**PC(18:2/18:2)**	1.08602	0.598696
**Mode -_PE(P-18:0/20:4)**	**PE(P-18:0/20:4)**	1.84461	-1.88698
**Mode -_PE(20:1/20:4)**	**PE(20:1/20:4)**	1.27511	-0.663051
**Mode -_PE(18:0/20:4)**	**PE(18:0/20:4)**	1.19903	-0.579199
**Mode -_PE(22:6/18:1)**	**PE(22:6/18:1)**	0.83121	-0.292808
**Mode +_PE(18:0/22:6)**	**PE(18:0/22:6)**	0.826663	-0.284938

**Mode -_PE(O-18:0/22:6)**	**PE(O-18:0/22:6)**	0.868883	-0.283409
**Mode -_PE(P-18:0/20:2)**	**PE(P-18:0/20:2)**	0.745452	-0.277779
**Mode -_PE(P-16:0/20:4)**	**PE(P-16:0/20:4)**	0.981789	-0.273066
**Mode +_PE(P-18:0/22:4)**	**PE(P-18:0/22:4)**	0.860013	-0.24005
**Mode -_PE(22:4/18:0)**	**PE(22:4/18:0)**	1.03667	-0.183936
**Mode +_PE(P-18:0/22:6)**	**PE(P-18:0/22:6)**	0.846771	-0.178836
**Mode +_PE(P-18:0/18:2)**	**PE(P-18:0/18:2)**	0.761173	-0.156093
**Mode -_PE(P-18:1/22:6)**	**PE(P-18:1/22:6)**	0.819117	-0.134644
**Mode -_PE(P-16:0/22:4)**	**PE(P-16:0/22:4)**	0.60781	-0.0282253
**Mode -_PE(16:0/22:6)**	**PE(16:0/22:6)**	0.600058	-0.0257649
**Mode -_PE(P-16:0/20:3)**	**PE(P-16:0/20:3)**	0.475244	-0.0194954
**Mode -_PE(16:0/20:4)**	**PE(16:0/20:4)**	0.545915	-0.0113733
**Mode -_PE(20:5/16:0)**	**PE(20:5/16:0)**	0.511122	-0.00632101
**Mode -_PE(O-18:0/18:2)**	**PE(O-18:0/18:2)**	0.313797	-0.00134008
**Mode +_PE(P-18:0/18:1)**	**PE(P-18:0/18:1)**	0.418147	-0.00126135
**Mode -_PE(P-16:0/18:1)**	**PE(P-16:0/18:1)**	0.366903	0.00425098
**Mode +_PE(P-16:0/22:6)**	**PE(P-16:0/22:6)**	0.864355	0.0463814
**Mode -_PE(16:0/18:2)**	**PE(16:0/18:2)**	0.805084	0.0479905
**Mode -_PE(20:5/18:1)**	**PE(20:5/18:1)**	0.464043	0.0970365
**Mode -_PE(O-18:0/18:1)**	**PE(O-18:0/18:1)**	0.76875	0.126085
**Mode -_PE(16:0/18:3)**	**PE(16:0/18:3)**	0.72128	0.171088
**Mode +_PE(18:1/18:1)**	**PE(18:1/18:1)**	0.851104	0.193085
**Mode +_PE(18:1/17:0)**	**PE(18:1/17:0)**	1.19235	0.226928
**Mode +_PE(34:2)**	**PE(34:2)**	1.05772	0.305057
**Mode +_PE(16:1/16:0)**	**PE(16:1/16:0)**	1.23515	0.316384
**Mode -_PE(18:1/18:2)**	**PE(18:1/18:2)**	0.858554	0.347899
**Mode +_PE(P-16:0/18:2)**	**PE(P-16:0/18:2)**	0.927682	0.364486
**Mode -_PE(18:2/18:2)**	**PE(18:2/18:2)**	1.37595	1.20309
**Mode -_PE(O-16:0/18:1)**	**PE(O-16:0/18:1)**	1.54041	1.42284
**Mode -_PG(18:1/18:1)**	**PG(18:1/18:1)**	0.648168	0.140006
**Mode -_PI(18:1/22:4)**	**PI(18:1/22:4)**	1.02442	-0.563268
**Mode -_PI(18:0/20:4)**	**PI(18:0/20:4)**	0.670739	-0.13245
**Mode -_PI(20:4/16:0)**	**PI(20:4/16:0)**	0.414641	-0.0514465
**Mode -_PI(18:1/18:0)**	**PI(18:1/18:0)**	0.357698	-0.0285417
**Mode -_PI(18:2/16:0)**	**PI(18:2/16:0)**	0.186046	-0.0181759
**Mode -_PI(16:0/18:1)**	**PI(16:0/18:1)**	0.575153	-0.00255493
**Mode -_PS(22:0/16:0)**	**PS(22:0/16:0)**	0.691227	-0.177542
**Mode -_PS(20:2/20:4)**	**PS(20:2/20:4)**	0.782813	-0.0855022
**Mode -_PS(18:1/20:2)**	**PS(18:1/20:2)**	0.671716	-0.0314262
**Mode -_PS(16:0/20:0)**	**PS(16:0/20:0)**	0.343293	-0.0174127
**Mode -_PS(18:2/20:2)**	**PS(18:2/20:2)**	0.690672	-0.00114038
**Mode -_PS(18:2/18:2)**	**PS(18:2/18:2)**	0.734935	0.00457329
**Mode -_PS(18:2/20:3)**	**PS(18:2/20:3)**	0.661247	0.00869048
**Mode -_PS(18:2/18:1)**	**PS(18:2/18:1)**	0.799902	0.0210943
**Mode -_PS(16:1/20:0)**	**PS(16:1/20:0)**	0.857765	0.431184
**Mode +_SM(34:2)**	**SM(34:2)**	1.28355	-0.70344
**Mode +_SM(41:1)**	**SM(41:1)**	1.05917	-0.299341
**Mode +_SM(d18:1/24:0)**	**SM(d18:1/24:0)**	1.01297	-0.297593
**Mode +_SM(38:3)**	**SM(38:3)**	1.05365	-0.248205

**Mode +_SM(d18:2/18:0)**	**SM(d18:2/18:0)**	0.866587	-0.126997
**Mode +_SM(40:1)**	**SM(40:1)**	1.00926	-0.100266
**Mode +_SM(d18:1/18:0)**	**SM(d18:1/18:0)**	1.01896	-0.0952459
**Mode +_SM(30:1)**	**SM(30:1)**	0.465453	-0.0816061
**Mode +_SM(33:1)**	**SM(33:1)**	0.96581	-0.0372163
**Mode +_SM(d18:1/19:0)**	**SM(d18:1/19:0)**	0.291112	-0.0141118
**Mode +_SM(d18:2/25:0)**	**SM(d18:2/25:0)**	0.806042	-0.00452279
**Mode +_SM(d18:1/20:0)**	**SM(d18:1/20:0)**	0.891011	0.000274332
**Mode +_SM(41:2)**	**SM(41:2)**	0.85766	0.00154643
**Mode +_SM(36:3)**	**SM(36:3)**	0.641322	0.00318869
**Mode +_SM(d18:2/24:0)**	**SM(d18:2/24:0)**	0.88299	0.00527447
**Mode +_SM(42:3)**	**SM(42:3)**	0.942605	0.00628839
**Mode +_SM(d18:1/22:1)**	**SM(d18:1/22:1)**	0.986452	0.0850724
**Mode +_SM(32:0)**	**SM(32:0)**	1.20336	0.496626
**Mode +_SM(36:0)**	**SM(36:0)**	1.68324	1.3433
**Mode +_SM(34:0)**	**SM(34:0)**	1.77878	1.43733
**Mode +_TG(13:0/16:1/18:1)**	**TG(13:0/16:1/18:1)**	0.561331	-0.0841042
**Mode +_TG(15:0/15:0/17:1)**	**TG(15:0/15:0/17:1)**	0.485565	-0.00982397
**Mode +_TG(16:1/18:1/22:0)**	**TG(16:1/18:1/22:0)**	0.435018	-0.00571819
**Mode +_TG(14:0/14:0/16:0)**	**TG(14:0/14:0/16:0)**	0.693073	0.00290237
**Mode +_TG(15:0/16:0/18:0)**	**TG(15:0/16:0/18:0)**	0.732489	0.0695474
**Mode +_TG(16:0/16:0/17:1)**	**TG(16:0/16:0/17:1)**	0.847747	0.0766748
**Mode +_TG(16:1/17:0/18:1)**	**TG(16:1/17:0/18:1)**	0.652062	0.0867277
**Mode +_TG(18:0/18:1/18:2)**	**TG(18:0/18:1/18:2)**	0.845864	0.11602
**Mode +_TG(16:1/18:1/18:1)**	**TG(16:1/18:1/18:1)**	0.402852	0.116096
**Mode +_TG(16:0/18:1/19:1)**	**TG(16:0/18:1/19:1)**	0.596401	0.138352
**Mode +_TG(18:0/18:1/18:1)**	**TG(18:0/18:1/18:1)**	0.83413	0.138426
**Mode +_TG(18:0/18:0/18:1)**	**TG(18:0/18:0/18:1)**	0.637459	0.156912
**Mode +_TG(15:0/15:0/16:1)**	**TG(15:0/15:0/16:1)**	0.982314	0.165719
**Mode +_TG(16:0/18:0/18:1)**	**TG(16:0/18:0/18:1)**	0.831046	0.170945
**Mode +_TG(14:0/16:0/16:0)**	**TG(14:0/16:0/16:0)**	1.0114	0.308247
**Mode +_TG(16:0/18:1/18:1)**	**TG(16:0/18:1/18:1)**	0.954098	0.310734
**Mode +_TG(14:1/16:1/18:1)**	**TG(14:1/16:1/18:1)**	1.15182	0.325341
**Mode +_TG(16:0/16:0/18:1)**	**TG(16:0/16:0/18:1)**	1.04125	0.471379
**Mode +_TG(18:1/18:1/18:2)**	**TG(18:1/18:1/18:2)**	1.12036	0.504664
**Mode +_TG(14:0/16:0/18:1)**	**TG(14:0/16:0/18:1)**	1.30893	0.637547
**Mode +_TG(16:0/16:1/18:2)**	**TG(16:0/16:1/18:2)**	1.39206	0.701532
**Mode +_TG(16:0/16:1/16:1)**	**TG(16:0/16:1/16:1)**	1.47398	0.761618
**Mode +_TG(16:0/16:1/18:1)**	**TG(16:0/16:1/18:1)**	1.56985	0.925374

Table S2. The 185 lipids identified in the serum of the broilers from the pHu line. The importance of each lipids in the model (VIP) and their contributions to the pHu+ (negative values) or pHu– (positive values) line modelized by OPLS-DA (M1a; 1 predictive and 3 orthogonal components, $R^2Y_{(cum)} = 0.74$ and $Q^2_{(cum)} = 0.56$) were tabulated in the third and fourth column, respectively.

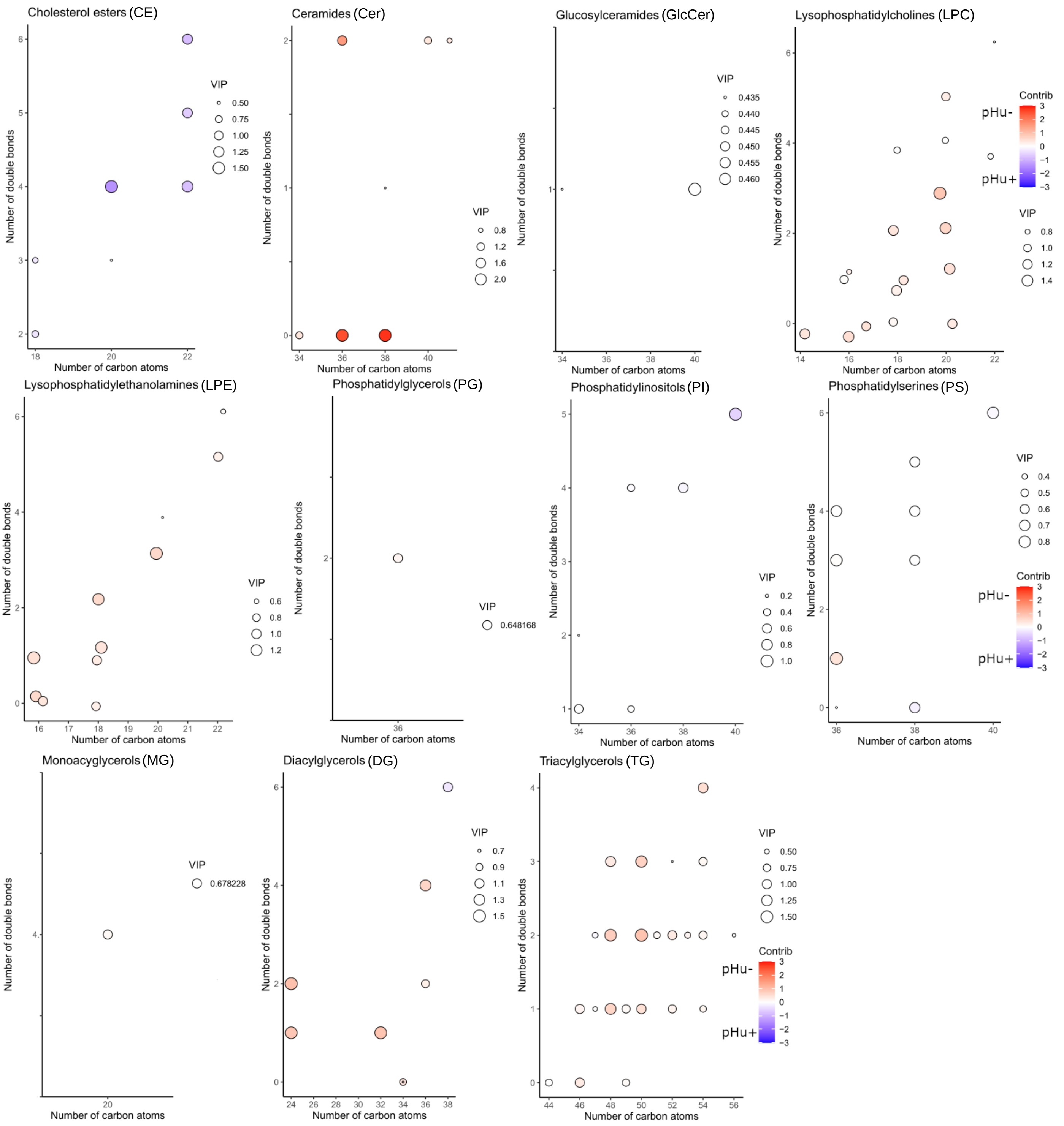

Figure S2. Relation between pHu lines, total number of carbon atoms and degree of unsaturation of acyl chains of the lipids. The importance of the lipids in the OPLS-DA model M1a (VIP) was represented by the size of the circle and the contribution to the pHu– or pHu+ lines was visualized through a gradient going from red (pHu–) to blue (pHu+). The characteristics of the M1a OPLS-DA model are 1 predictive and 3 orthogonal components, $R^2Y_{(cum)} = 0.74$ and $Q^2_{(cum)} = 0.56$. Position jitter was introduced in the LPC and LPE plots to avoid overplotting.

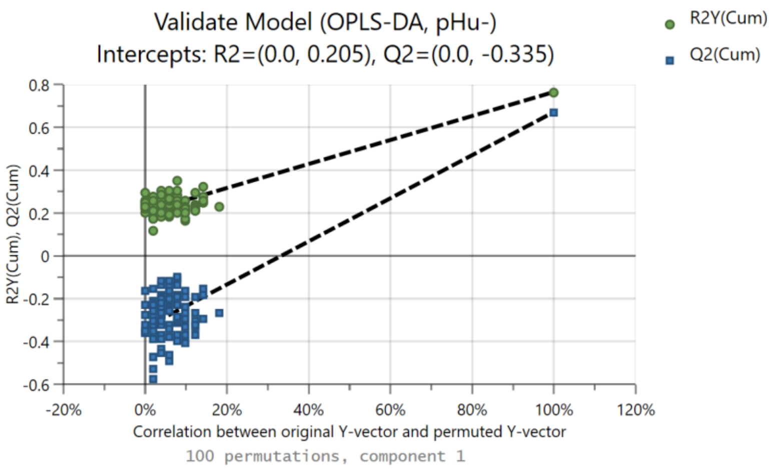

Figure S3. Permutation plot for the OPLS-DA model M1b (pHu lines). The permutation plot check the validity and the degree of overfit for the model. The plot displays the correlation coefficient between the original y-variable (i.e. pHu+ or pHu–) and the permuted y-variable on the x-axis versus the cumulative R^2 and Q^2 on the y-axis, and draws the regression line. The intercept is a measure of the overfit.

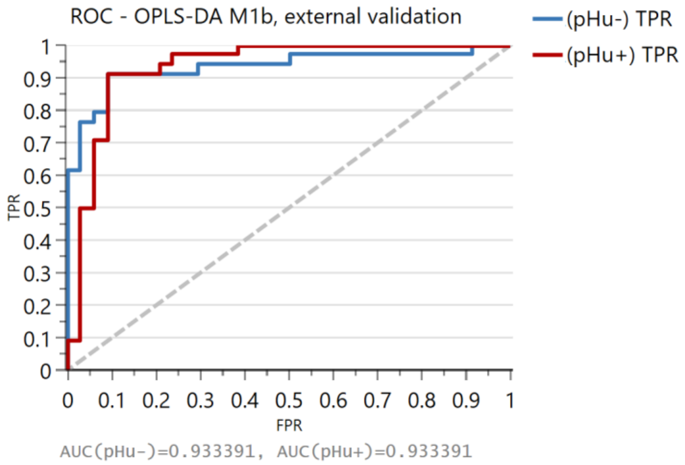

Figure S4. ROC curve external validation OPLS-DA model M1b (pHu lines). The ROC curve is a tool for visualizing and summarizing the performance of classification and discrimination models. The plot displays the true positive classification rate (TPR) of a classifier model plotted against the corresponding false positive classification rate (FPR) at various threshold settings of the criterion parameter Y predicted.

As a quantitative measure of the classification success the area under the ROC curve (AUC) is computed. This parameter ranges between 0.5 (bad classification) and 1.0 (perfect classification).

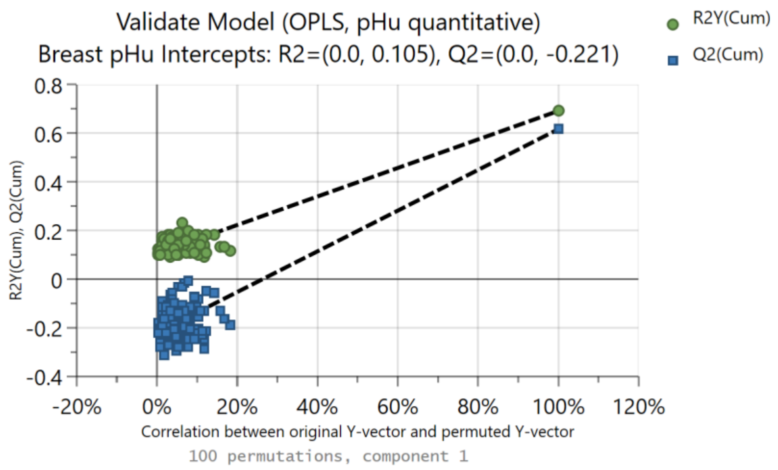

Figure S5. Permutation plot for the OPLS model M4 (pHu value). The permutation plot check the validity and the degree of overfit for the model. The plot displays the correlation coefficient between the original y-variable (i.e. pHu) and the permuted y-variable on the x-axis versus the cumulative R^2 and Q^2 on the y-axis, and draws the regression line. The intercept is a measure of the overfit.

Model OPLS-DA (Line; M1b)	VIP
Mode +_Cer(d18:0/20:0)	1.85969
Mode -_PE(P-18:0/20:4)	1.81344
Mode +_Cer(d18:0/18:0)	1.72967
Mode +_CE(20:4)	1.5651
Mode +_TG(16:0/16:1/18:1)	1.47464
Mode +_Cer(d18:1/18:1)	1.47063
Mode +_SM(34:0)	1.39926
Mode -_PE(O-16:0/18:1)	1.39021
Mode +_LPC(20:3)	1.37123
Mode +_SM(36:0)	1.26038
Mode -_PE(18:2/18:2)	1.25731
Mode +_CE(22:6)	1.235
Mode -_PE(20:1/20:4)	1.21721
Mode +_LPC(16:0)	1.2061
Mode +_SM(34:2)	1.1958
Mode +_PE(16:1/16:0)	1.18038
Mode +_LPC(18:1)	1.1636
Mode -_PI(18:1/22:4)	1.13942
Mode +_LPC(17:0)	1.12518
Mode +_TG(18:1/18:1/18:2)	1.11379
Mode +_Cer(d18:0/16:0)	1.02471
Mode -_PE(22:4/18:0)	1.01434
Mode -_PC(18:0/18:1)	1.00386
Mode +_DG(18:2/20:4)	1.00089
Mode +_LPC(16:1)	0.998122
Mode +_SM(d18:1/24:0)	0.996711
Mode +_PE(34:2)	0.983252
Mode +_SM(38:3)	0.974077
Mode +_PC(O-22:0)	0.957509
Mode +_LPC(20:4)	0.942643
Mode +_CE(18:2)	0.922736
Mode +_PC(O-34:0)	0.895652
Mode +_SM(42:3)	0.889393
Mode +_PC(32:2)	0.887753
Mode +_CE(18:3)	0.886779
Mode -_PS(18:1/20:2)	0.878176
Mode -_PC(18:1/22:6)	0.877409
Mode -_PE(16:0/18:2)	0.872093
Mode +_PC(18:1/19:0)	0.832525
Mode -_PS(16:1/20:0)	0.82469
Mode +_LPC(18:4)	0.821205
Mode +_TG(16:0/18:0/18:1)	0.818968
Mode +_SM(d18:2/18:0)	0.814218
Mode -_PE(P-18:0/20:2)	0.802684
Mode +_PE(P-18:0/18:2)	0.795111
Mode +_Cer(d18:2/23:0)	0.752075
Mode +_TG(18:0/18:1/18:1)	0.747223
Mode +_PE(P-16:0/22:6)	0.72382
Mode -_LysoPE(22:5)	0.719258
Mode +_PE(P-16:0/18:2)	0.713559
Mode -_PC(O-16:0/22:5)	0.707875
Mode -_PS(18:2/18:2)	0.706873
Mode +_TG(18:0/18:0/18:1)	0.698659
Mode +_PC(O-18:0/18:2)	0.676071
Mode -_PE(P-16:0/22:4)	0.675426
Mode +_SM(d18:1/20:0)	0.674651
Mode +_Cer(d18:1/20:0)	0.645368
Mode +_DG(18:0/16:0)	0.634939
Mode -_PC(O-18:1/18:2)	0.618063
Mode +_TG(16:0/18:1/19:1)	0.617894
Mode +_TG(13:0/16:1/18:1)	0.602118
Mode -_PC(18:0/20:2)	0.584381
Mode -_PE(16:0/20:4)	0.579373
Mode +_GlcCer(d18:1/22:0)	0.573233
Mode -_PE(20:5/18:1)	0.494819
Mode +_SM(30:1)	0.480349
Mode +_TG(16:1/18:1/18:1)	0.472926
Mode -_PS(16:0/20:0)	0.360961

Model OPLS (pHu value; M4)	VIP
Mode +_Cer(d18:0/20:0)	1.81235
Mode +_Cer(d18:0/18:0)	1.64782
Mode -_PE(P-18:0/20:4)	1.5795
Mode +_SM(34:0)	1.54753
Mode +_SM(36:0)	1.34
Mode +_LPC(20:3)	1.32623
Mode -_PE(18:2/18:2)	1.31264
Mode +_CE(20:4)	1.24836
Mode +_LPC(18:1)	1.18537
Mode +_Cer(d18:1/18:1)	1.18377
Mode +_Cer(d18:0/16:0)	1.13775
Mode +_LPC(17:0)	1.1304
Mode -_PE(O-16:0/18:1)	1.06487
Mode +_LPC(16:1)	1.06285
Mode +_TG(18:1/18:1/18:2)	1.02736
Mode +_PC(O-34:0)	0.964985
Mode +_SM(d18:2/18:0)	0.902723
Mode +_LPC(18:4)	0.898271
Mode +_PC(O-18:0/18:2)	0.891276
Mode +_SM(d18:1/20:0)	0.883637
Mode -_PE(20:1/20:4)	0.879974
Mode -_PC(18:1/22:6)	0.875134
Mode +_PC(32:2)	0.860301
Mode +_PE(P-16:0/22:6)	0.827999
Mode +_PE(P-18:0/18:2)	0.813128
Mode -_PC(18:0/18:1)	0.810042
Mode -_PS(16:1/20:0)	0.807613
Mode +_PE(P-16:0/18:2)	0.746543
Mode +_TG(18:0/18:1/18:2)	0.740285
Mode -_PE(P-16:0/20:3)	0.650669
Mode +_DG(18:0/16:0)	0.646717
Mode +_TG(16:0/18:1/19:1)	0.611252
Mode -_LysoPE(22:5)	0.59722
Mode +_PC(18:1/19:0)	0.568204
Mode +_TG(13:0/16:1/18:1)	0.543889
Mode -_PC(O-18:1/18:2)	0.543648
Mode +_CE(18:2)	0.525974
Mode -_PC(16:1/20:4)	0.505816
Mode +_CE(18:3)	0.469835
Mode +_TG(16:1/18:1/18:1)	0.410329

Table S3. Comparison between the lipid sets included in the model OPLS-DA predictive of the pHu lines (M1b) and the model OPLS predictive of the pHu value (M2). The lipids were ordered according their importance in the model (VIP) and highlighted in red when they were present in both models.
